# A study of cardiac complications in HIV infected children

**DOI:** 10.1186/1471-2334-14-S3-O24

**Published:** 2014-05-27

**Authors:** S Pushpalatha, K Anitha

**Affiliations:** 1 Department of Pediatrics, Bangalore Medical College and Research Institute, Bangalore, India

## Background

Studies have demonstrated the presence of abnormality on echocardiography in HIV infected adults, but there is a paucity of similar data regarding children in the Indian subcontinent. The objectives of this study were to evaluate the cardiac abnormalities in HIV infected children and to know the metabolic and nutritional risk factors for cardiac disease.

## Methods

One hundred consecutive HIV infected children were subjected to history, examination, nutritional assessment, complete hemogram, lipid profile,CD4 count, chest X ray, 12 lead ECG and M mode transthoracic 2D ECHO. Statistical analysis was done using uni variate and multi variate regression analysis.

## Results

Out of 100 HIV infected children, 42% [25 males, 17 females] had cardiac abnormalities of which only 6 [14%] were symptomatic. Majority belonged to WHO clinical stage IV, with 13 [28%] having low CD4 count. The most common cardiac abnormality was left ventricular systolic dysfunction (LVSD) in 19% (*p*<0.05). Other abnormalities were mild tricuspid regurgitation in 14%, pericardial effusion in 6%, pulmonary artery hypertension in 6%, dilated right ventricle, right atrium in 3%, pulmonary regurgitation in 3% and mitral regurgitation in 1%. ECG showed sinus tachycardia in 14%, right ventricular hypertrophy in 6%, irregular rhythm in 3%. Among children with cardiac abnormalities, dyslipidemia was seen in 32 [77%] (*p*<0.05), anemia in 29 [69%](*p*<0.05),and malnutrition in 19[45%](*p*>0.05).

## Conclusion

Though the majority of the children were asymptomatic, investigations revealed 42% with subclinical cardiac abnormalities. They had significant dyslipidemia and anemia as compared to children without cardiac abnormalities. The study thus emphasizes the need for routine echocardiographic screening.

